# Structure Determination of Feline Calicivirus Virus-Like Particles in the Context of a Pseudo-Octahedral Arrangement

**DOI:** 10.1371/journal.pone.0119289

**Published:** 2015-03-20

**Authors:** Wim P. Burmeister, Marlyse Buisson, Leandro F. Estrozi, Guy Schoehn, Olivier Billet, Zahia Hannas, Cécile Sigoillot, Hervé Poulet

**Affiliations:** 1 Unit of Virus Host Cell Interactions, Université Grenoble Alpes, Grenoble, France; 2 Unit of Virus Host Cell Interactions, Unité Mixte Internationale 3265, Centre National de Recherche Scientifique, Grenoble, France; 3 Laboratoire de Virologie, Centre Hospitalo-Universitaire de Grenoble, Grenoble, France; 4 Institut de Biologie Structurale Jean-Pierre Ebel, Commissariat d’Energie Atomique, Grenoble, France; 5 Institut de Biologie Structurale Jean-Pierre Ebel, Centre National de Recherche Scientifique, Grenoble, France; 6 Institut de Biologie Structurale Jean-Pierre Ebel, Université Grenoble Alpes, Grenoble, France; 7 Lyon Gerland Laboratory, Merial R&D, Lyon, France; CSIC/CNB, SPAIN

## Abstract

The vesivirus feline calicivirus (FCV) is a positive strand RNA virus encapsidated by an icosahedral T=3 shell formed by the viral VP1 protein. Upon its expression in the insect cell - baculovirus system in the context of vaccine development, two types of virus-like particles (VLPs) were formed, a majority built of 60 subunits (T=1) and a minority probably built of 180 subunits (T=3). The structure of the small particles was determined by x-ray crystallography at 0.8 nm resolution helped by cryo-electron microscopy in order to understand their formation. Cubic crystals belonged to space group P2_1_3. Their self-rotation function showed the presence of an octahedral pseudo-symmetry similar to the one described previously by Agerbandje and co-workers for human parvovirus VLPs. The crystal structure could be solved starting from the published VP1 structure in the context of the T=3 viral capsid. In contrast to viral capsids, where the capsomers are interlocked by the exchange of the N-terminal arm (NTA) domain, this domain is disordered in the T=1 capsid of the VLPs. Furthermore it is prone to proteolytic cleavage. The relative orientation of P (protrusion) and S (shell) domains is alerted so as to fit VP1 to the smaller T=1 particle whereas the intermolecular contacts around 2-fold, 3-fold and 5-fold axes are conserved. By consequence the surface of the VLP is very similar compared to the viral capsid and suggests a similar antigenicity. The knowledge of the structure of the VLPs will help to improve their stability, in respect to a use for vaccination.

## Introduction

**Fig 1:**
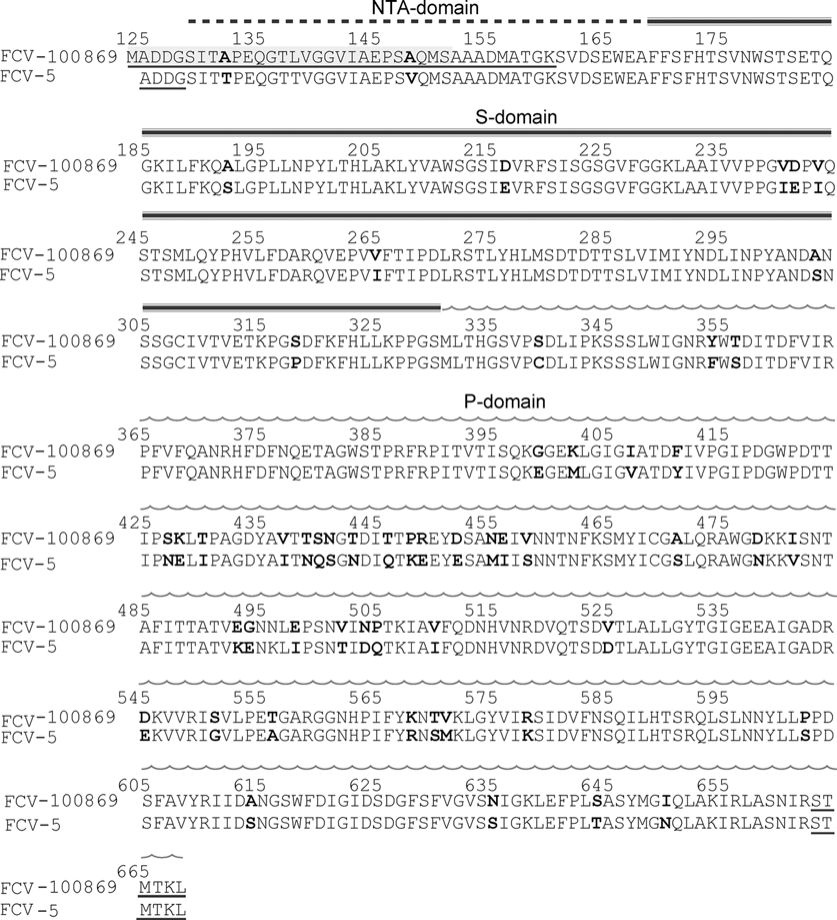
Sequence alignment of the VP1 sequence from our study (Merial100869, top) with the one (FCV-5) present in the crystal structure of FCV [2]. Residues differing between the two sequences are printed in bold. Residues for which electron density is missing are underlined. The peptide missing in a fraction of the recombinant VP1 molecules due to proteolysis is shaded in gray. The domain organization in NTA (dotted bar), S (triple line) and P domain (wavy line) is indicated above the sequence.

The *Vesivirus* feline calicivirus (FCV) belongs to the family of *Caliciviridae* which carry a positive strand RNA genome which is enclosed in a capsid composed of 180 copies of the major capsid protein forming an icosahedral T = 3 shell. High-resolution crystallographic or cryo-electron microscopy structures of several caliciviruses elucidated the capsid structure for the *Vesiviruses* San Miguel sea lion virus (SMSV) [1], FCV [2], the *Noroviruses* Norwalk virus (NV) [3] and murine norovirus (MNV) [4] and the *Lagovirus* rabbit hemorrhagic disease virus (RHDV) [5]. These structures showed that the major capsid protein (VP1 in vesiviruses) is composed of 3 domains: a short disordered peptide downstream of the proteolytic cleavage site (res. 125–128 in FCV) is followed by the N-terminal arm (NTA) domain (res. 129–173) interlocking with neighboring subunits for the formation of the capsid. The S domain which forms the shell (res. 174–330) is followed by the C-terminal P (protrusion) domain (res. 331–668) which forms a dimeric spike carrying the receptor binding site (Fig. 1). In contrast to the other genera, *Vesivirus* VP1 is synthesized as a precursor (668 residues for FCV) which is processed by the viral protease into a 124 res. leader peptide and the 544 res. VP1 protein. The leader peptide of the capsid was described to enhance the cytopathic effect of FCV [6]. Feline Junctional Adhesion Molecule-A (fJAM-A) was identified as a cellular receptor for FCV [7].

FCV infects mainly domestic cats where it can be unapparent or can be associated with a range of clinical syndromes such as mild oral and upper respiratory tract disease, with or without acute polyarthritis. FCV has become more problematic due to the recent appearance of hypervirulent strains causing virulent systemic disease (VSD), first described in California in 1998 [8]. These strains causing high mortality and morbidity motivate vaccination of pet cats. Currently both inactivated and live attenuated FCV vaccines are used. Recombinant FCV VP1 produced in insect cells using the baculovirus system was studied as vaccine candidate against FCV infection in domestic cats [9]. Our study was motivated by the need for understanding structure, formation and stability of VLPs produced for vaccine development.

We observed that the majority of the FCV VLPs obtained from the expression of VP1 in the baculovirus system are small sub-viral particles made of 60 subunits with T = 1 symmetry different from viral capsids. We determined their structure by x-ray crystallography helped by an initial reconstruction from cryo-electron microscopy. The structure allowed us to understand the structural changes of VP1 required to switch from T = 3 to T = 1 symmetry. On the way to the x-ray structure we had to overcome obstacles arising from an octahedral pseudo-symmetry of the VLP’s evoking a historical controversy on the symmetry of viral particles [10, 11]. As an octahedral self-organization of the recombinant protein in the context of the crystal could not be excluded in beforehand, the pseudo-symmetry was particularly misleading.

## Materials and Methods

### VLP production and purification

The sequence of FCV strain Merial100869 coding for mature VP1 (res. 125–668) was codon-optimized for insect cells (Geneart, Germany). This synthetic DNA was used to generate a recombinant baculovirus for protein expression under the polyhedrin promoter by homologous recombination. The sequence starts with the methionine residue of the start codon introduced before the first residue of VP1 at position 124 (Fig. 1). Briefly, *Spodoptera frugiperda* 9 (Sf 9) insect cells were co-transfected with a plasmid containing the VP1 sequence and with Bsu36I triple-cut linearized AcNPV DNA, according to the manufacturer’s protocol (Baculogold, Pharmingen). Recombinant baculovirus from co-transfection supernatants was plaque-purified twice. Five clones were amplified at 27°C in 25 cm² monolayer flasks. Infected cells and supernatants were analyzed for FCV VP1 expression by Western blot using a monoclonal antibody (Antibody H3-21012E22E, Merial). The best clone was further amplified at 27°C at the 50 ml scale in Erlenmeyer flasks under agitation at 105 rpm. A third passage at the 200 ml scale was performed in order to obtain the virus stock used for protein expression. FCV VLPs were produced in a 7 l bioreactor (Applikon) using serum free medium. After infection of Sf9 cells (concentration 10^6^ cells/ml) and culture for 6 days post-infection, the supernatant containing VLP’s was harvested by centrifugation. Supernatants were concentrated using an Amicon Ultra 4 100k (Millipore) device. VLPs were purified over a 30% sucrose cushion by centrifugation at 30 000 rpm in a SW 32Ti rotor at 4°C for 16 h. Proteins were dialyzed to 20 mM MES pH 6, 200 mM NaCl and concentrated for crystallization to 3.4 mg·ml^-1^ in an Amicon Ultra 4 100k.

### Electron microscopy

For negative stain electron microscopy (EM), samples were applied to the clean surface of a carbon film deposited on a mica surface and stained with 2% ammonium molybdate. Electron micrographs were taken with a TEM CM12 120 kV LaB6 (Philips) equipped with an Orius 2.6k×4k CCD camera (Gatan).

For cryo-EM, the sample was concentrated five times using a 100 kDa cutoff Centricon (Millipore). 4 μl of sample were loaded onto a Quantifoil R2/1 holey grid (Quantifoil Micro Tools GmbH, Germany) and vitrified using a Mark IV vitrobot (FEI). The frozen grid was transferred to a Polara electron microscope working at 300 kV. Images were taken under low-dose conditions (less than 20 e^-^/Å^2^) and with a nominal magnification of 39,000× on KODAK SO-163 films. Negatives were developed in full strength D19 developer for 12 min.

### Image analysis

Negatives were screened for astigmatism and drift by optical diffraction. 20 negatives were digitized using a Photoscan TD scanner with a pixel size of 7 μm (1.79 Å per pixel at the specimen level).

The contrast transfer function (CTF) was determined for each micrograph using the ctffind3 program [12] and the micrographs were CTF-flipped by the bctf program from the Bsoft package [13]. An *ab-initio* 3D model was obtained with the software RIco [14] by using a set of 30 manually selected particles from a 4-fold binned micrograph with 3.0 μm underfocus. By following the protocol described in Estrozi & Navaza [15] we refined the initial model using 11158 particles that were semi-automatically picked by the boxer program [16]. The Fourier-shell correlation (FSC) shown in S1 Fig. using a threshold of 0.5 estimates the resolution of the final 3D reconstruction to 14 Å [17], certainly due to sample heterogeneity and/or flexibility. The model was deposited in the EMDataBank under accession number 2823.

The atomic model of a VP1 monomer [2] was fitted into the EM reconstruction using the URO software [18] resulting in a (maximized) correlation coefficient between the atomic model and the map of 0.62 for data up to 10 Å resolution.

### Crystallization and data collection

First crystals were obtained at the nanodrop crystallization facility of the EMBL-PSB using a Cartesian robot and 100 + 100 nl drops. Crystallization conditions were manually refined using the hanging drop method leading to a reservoir solution of 7–10% PEG 6000, 0.1 M Hepes pH 7, 0.5–1 M LiCl. Octahedron- or tetrahedron-shaped crystals grew within a few days. Crystals were cryoprotected briefly in a mix of 80% reservoir solution and 20% (v/v) glycerol and frozen in liquid nitrogen. 8 Å data were collected at 100 K on beamline ID23-2 at the European Synchrotron Radiation Facility in Grenoble, France, using an ADSC Quantum4 detector. Data were processed using iMOSFLM [19] and SCALA.

### Crystallographic structure determination

Self-rotation functions were calculated with MOLREP [20] and GLRF [21]. Translation functions were calculated with AMORE [22]. Different programs of the ccp4 program suite were used, in general through ccp4i [23]. Models were built using PyMol (www.pymol.org) and coot [24]. In order to generate icosahedral models, the structure of monomer C from pdb entry 3m8l [2] was manipulated with coot and expanded with pdbset to the full icosahedron using matrices obtained from http://viperdb.scripps.edu. Correlation and R-factor searches in real space (mainly in the 15–8 Å resolution range) used 1/3 of the VLP forming the asymmetric unit (20 monomers) and a linux script combining refmac [25] of the ccp4 package for structure factor calculation and moleman of the Uppsala software factory programs (http://xray.bmc.uu.se/usf/) for coordinate translations.

Once the model was positioned in the asymmetric unit (asu), the 20-mer model of the asymmetric unit was refined as one rigid-body. Non-crystallographic symmetry (ncs) operators were calculated from the 20-mer structure using CNS. Rigid-body refinement of one VP1 subunit imposing strict 20-fold ncs used CNS [26]. The refined subunit structure was expanded to a full asu with pdbset. Rigid-body refinement of the 20-mer and of an individual subunit were alternated; at later steps position and orientation of S and the P domains were refined separately, always imposing 20-fold ncs. The calculated sigmaA-weighted electron density was averaged in dm [27] using 20-fold ncs. When it became obvious that the P domain did not match the electron density, it was positioned by hand and using real space rigid-body refinement in coot. At the same time we noticed that there was no electron density for the N-terminal 36 residues which we deleted subsequently. In a final step the sequence was corrected to the one of strain Merial100869 present in our construct using the mutagenesis tool of coot and standard rotamers for new side chains. Individual atom positions were not refined, except for mutations to proline residues where the chain was regularized. The temperature factor of the structure was set to 427 Å^2^, the value obtained from Xtriage [28]. The structure is deposited in the pdb (entry 4pb6).

### Crystallographic full restrained refinement

The rigid body refined model of the 20 subunits forming the asu described in the previous section was submitted to constrained refinement using refmac5, preceded by TLS refinement of the P domain (res. 331–662) as, justified by the electron density of the averaged map where the P domains appear weaker than the S domains indicating disorder. 190 ncs constraints between the 20 subunits were generated automatically by the refmac5 program. Tight constraints were used at the level of position and temperature factor. Final statistics are given in S1 Table.

## Results and Discussion

### Production of FCV VLPs

**Fig 2:**
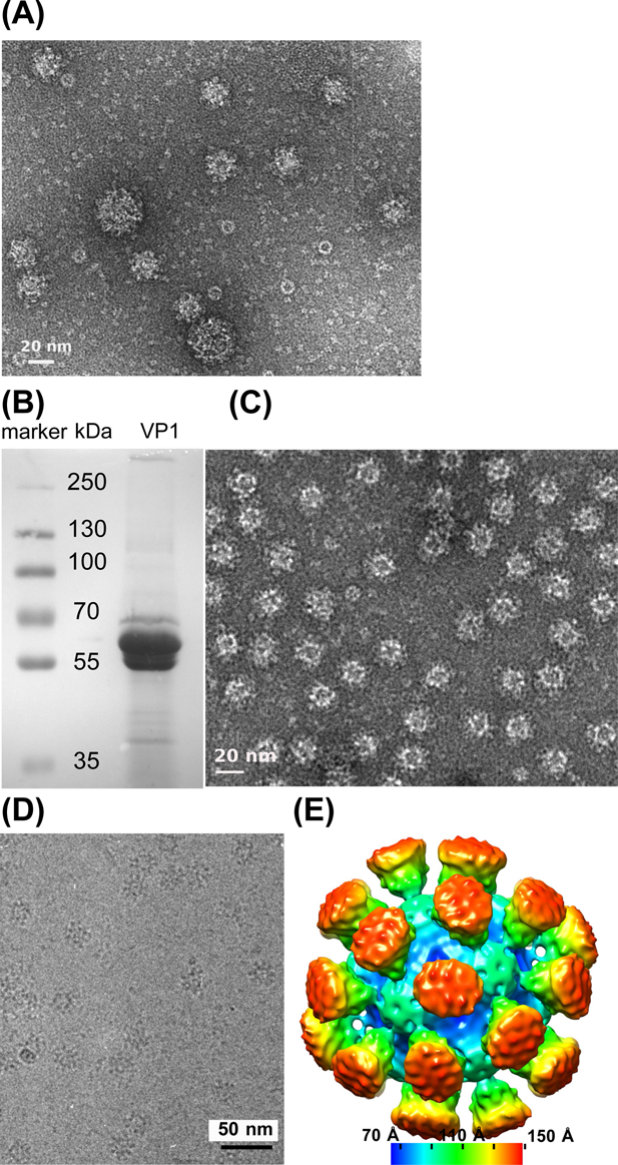
FCV VLPs. (A) Negative stain electron microscopy of FCV VLP particles from insect cell culture supernatants. Note the presence of some large particles, which correspond probably to a T = 3 arrangement of VP1 capsomers, besides a majority of smaller VLPs with T = 1. Monomeric or dimeric VP1 and some ring-like contaminant from the expression system are also present. (B) SDS PAGE of sucrose gradient purified VLPs. VP1 runs always as a doublet where the smaller form has been proteolyzed N-terminally (see Fig. 1). (C) Sucrose gradient purified VLPs (T = 1) in negative stain. (D) Cryo-EM image of the same VLPs. (E) Low-resolution EM reconstruction using icosahedral symmetry of the T = 1 VLPs viewed in cryo-EM as shown in panel (E). The surface is colored according to the distance from the particle’s center.

Supernatants from baculovirus-infected insect cells showed two forms of VLPs, a majority with a diameter of 28 nm and a minority with a diameter of 40 nm besides some monomeric protein (Fig. 2A). As the viral particles of FCV have a diameter of 40 nm [2], we assumed that the big form corresponded to T = 3 icosahedral VLPs composed of 180 molecules of VP1 whereas the small form corresponded to T = 1 VLPs composed of 60 subunits. After purification on a sucrose cushion, VLPs were recovered in the bottom fraction. The fraction contained mainly VP1 which migrated as two bands on a SDS-PAGE gel with apparent molecular weights of 55 kDa and 60 kDa (Fig. 2B). N-terminal sequencing indicated that the form migrating at 55 kDa started at residue 152 (Fig. 1, theoretical MW = 56.5 kDa) and the heavier one (theoretical MW = 59.2 kDa) corresponded to the full length construct. This was shown indirectly by the absence of a signal in N-terminal sequencing due to an acetylated N-terminus of the baculovirus expressed protein. Electron microscopy of the fraction showed the presence of small T = 1 VLPs (Fig. 2C, 2D). The low-resolution reconstruction by cryo-electron microscopy of the small VLPs confirmed an icosahedral structure in solution composed of 60 monomers (T = 1, Fig. 2E).

### Structure determination and refinement

**Fig 3:**
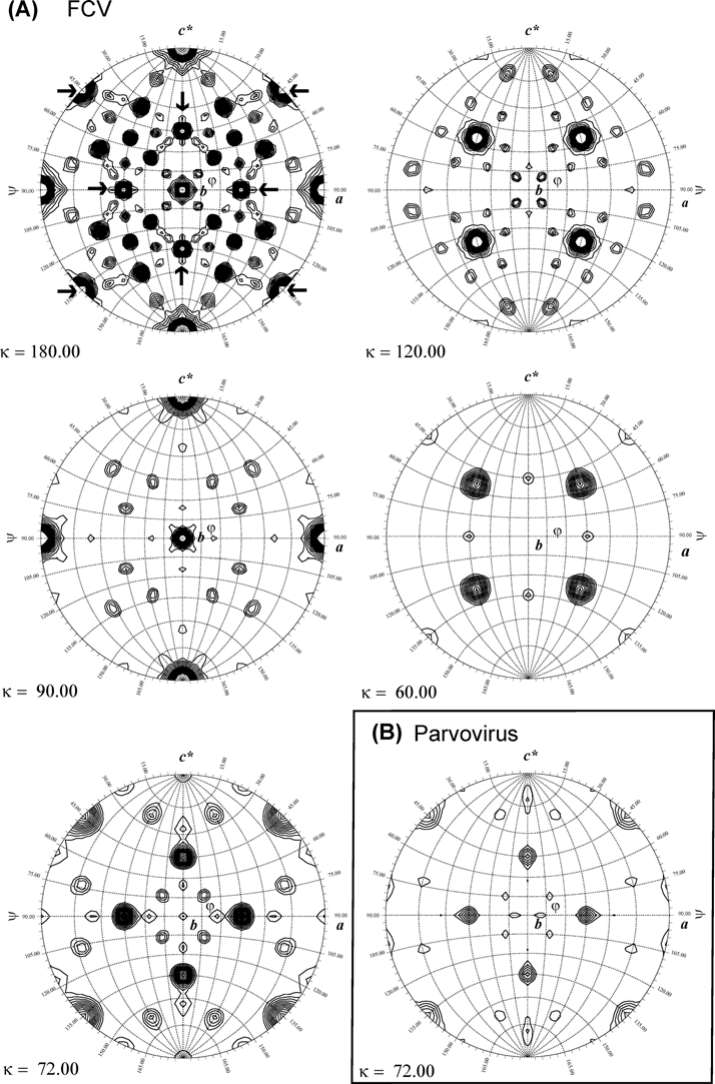
Self-rotation functions. (A) Self-rotation function of the FCV VP1 crystal plotted for different κ-sections. Plots are contoured at 1 σ with 0.5 σ increments. Data were used between 8 and 25 Å resolution with a radius of integration of 100 Å. (B) κ = 180° section of the self-rotation function of parvovirus crystals (pdb entry 1s58, [30]) calculated from deposited structure factors using the same parameters as in panel A. Ncs peaks appear lower than the ones in the corresponding section of panel A due to lower completeness of the parvovirus data.

The structure of the VP1 subunits was available from the crystal structure of the T = 3 viral capsid. A pseudo-atomic model of the FCV VLP was built based on the low-resolution electron density map of T = 1 VLPs obtained from cryo-EM (Fig. 2E) and the structure of VP1 (pdb entry 3m8l, [2]) of isolate FCV-5 [29] whose sequence is 89% identical to our isolate. Crystals of FCV VLPs diffracted to about 12 Å resolution but one partially dehydrated crystal from a badly sealed drop diffracted to 8 Å resolution and allowed the collection of a full dataset, data between 8 and 9 Å resolution still being weak (Table 1). The crystal belongs to the cubic space group P2_1_3 with a cell parameter of 358.3 Å. The presence of T = 3 particles could be excluded from packing considerations. The self-rotation function appeared to be rather complex (Fig. 3A) with the presence of 4-fold (κ = 90°) and 6-fold (κ = 60°) axes. The position of strong peaks in the κ = 72° sections suggesting 5-fold axes related by 4-fold symmetry suggested an octahedral symmetry and was particularly intriguing.

**Table 1:** Statistics on data collection, structure determination and refinement.

	Data collection
**Wavelength (Å)**	0.9185
**Resolution range (Å)**	67–8.0 (9.6–8.9)^a^ (8.4–8.0)
**No. of unique reflections**	13102 (1925) (752)
**Completeness (%)**	87.2 (88.7) (61.5)
**Redundancy**	5.5 (5.5) (1.8)
**< *I*/σ(*I*)>**	3.1 (1.3) (0.7)
***R***_**sym**_	0.240 (0.576) (1.10)
	**Structure determination and refinement**
**Resolution range (Å)**	67–8.0 (9.6–8.9) (8.4–8.0)
**R**_**cryst**_ **from refmac**	0.427 (0.41) (0.41)
**R**_**free**_ **from refmac**	0.448 (0.39) (0.41)
**Wilson-plot B-factor from Xtriage [28]**	427 Å^2^
**Electron density CC** ^**b**^ **after rigid-body refinement**	0.64
**Electron density CC** ^**b**^ **after averaging**	0.94
**Degrees of freedom in rigid-body refinement**	13^c^

^a^ As data are weak and incomplete in the 8 Å to 8.4 Å bin, statistics are also given for the 9.6–8.9 Å bin.

^b^ Correlation coefficient.

^c^ 6 degrees of freedom describe the overall position and orientation of the capsomer, 6 the changes of the relative orientation and position of the S and P domains and an overall temperature factor is refined.

As molecular replacement using the pseudo-atomic model was fruitless, we searched the Protein Data Bank (pdb) for crystals with the same space group and a similar unit cell. This led us to the structure of recombinant parvovirus B19 VP2 forming icosahedral VLPs with T = 1 (pdb entry 1s58, [30]). A comparison of the self-rotation functions of FCV and parvovirus VLP crystals showed a striking resemblance, in particular concerning the arrangement of 5-fold symmetry axes (Fig. 3A and 3B, κ = 72° section). It was very likely that the orientation of the VLPs would be similar in both crystals. The parvovirus VLP crystal structure contains 1/3 of the VLP in the asymmetric unit with an alignment of an icosahedral 3-fold axis with the (111) vector of the cubic P2_1_3 unit cell. The only remaining degrees of freedom are a κ-rotation around this vector and the translation along this direction (φ = 45°; ψ = 54.7°). For the parvovirus crystals the rotation corresponds to κ = -21.02° relative to an icosahedral capsid in standard orientation 2(z)-3-5-(x)2 [31].

The exact orientation of the FCV VLP and its position along the (111) vector still had to be determined. Parvovirus capsids are smooth, almost spherical particles where the position could be inferred from simple packing considerations [32]. In case of FCV, the packing is less obvious due to the presence of prominent spikes (Fig. 2E) which might interlock leading to a position of the particle different from the (0.25,0.25,0.25) position of smooth spheres.

**Fig 4:**
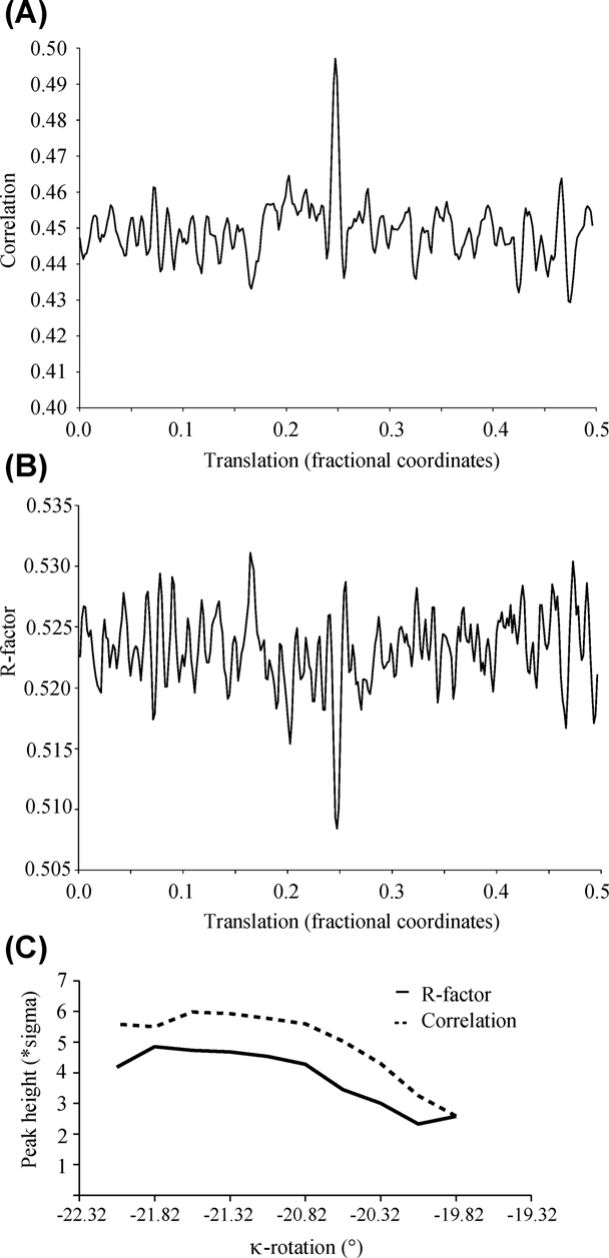
Search for the position of the VLP in the asymmetric unit. (A) Translation search. The calculated correlation between observed and calculated structure factor amplitudes in the 15 to 8 Å range in function of the translation along the (111) diagonal of the model, initially centered on the origin, is plotted. The search model is rotated by κ = -23.82 relative to the standard orientation which led to the best solution (see panel C). (B) The corresponding plot of the R-factor in function of the translation. (C) Plot of the peak heights of the normalized correlation coefficient (as in panel A) or R-factor (as in panel B) at (0.25,0.25,0.25) in function of the κ-rotation. No significant peak was obtained for κ = -19.32°.

In order to position the particle, a new model of FCV VLPs had been built manually from the structure of the VP1 monomer using the constraint of the best possible conservation of the contacts around the 5-fold axis, around the 3-fold axis and around the 2-fold axis. In order to fulfill these constraints, the relative angle and position of the P domains relative to the S domains had to be modified. A correlation and R-factor search was used which translated the model covering one asu along the (111) vector. This one-dimensional search was carried out for several models where the κ-angle was changed in steps of 0.5° from an initial orientation of κ = -19.32°. This approach allowed to position the model of the asymmetric unit unambiguously (Fig. 4) using a translation of (0.25,0.25,0.25) and κ = -23.82°.

This model has been improved further by rigid-body refinement. It became obvious that here was no electron density for the N-terminal 37 residues which correspond roughly to the NTA domain (Fig. 1). In the T = 3 capsid these residues are involved in inter-subunit contacts interlocking the shell of VP1 capsid proteins. These residues were excluded from the model and as they are either disordered or proteolyzed. After additional cycles of rigid-body refinement the R_cryst_ decreased to 0.451 in the resolution range between 8 Å and 67 Å and the electron density map showed an average correlation coefficient of 0.64 between ncs related subunits which increased to 0.94 after density improvement by ncs averaging and solvent flattening (Table 1). The final model has been adjusted for the sequence of the Merial100869 strain and yields an R_cryst_ of 0.427 and an R_free_ of 0.451 in the 8 Å and 67 Å resolution range. Although the information provided by the 8 Å electron density map was limited, there was no indication of additional electron density corresponding to the N-terminal residues.

The final orientation of the VLP compared to icosahedral standard orientation corresponds to κ = -23.03° close to the κ = -21.02° orientation of the parvovirus particle in its high resolution structure [30]. An approximate alignment of a 2-fold axis of the icosahedron with the crystallographic (110) direction results from this orientation of the particle (Fig. 3A, 180° section, arrows). Together with the 2-fold crystallographic screw axis along the (100) direction this generates a 4-fold ncs around a direction perpendicular to (110) and (100) which corresponds to the (001) direction (Fig. 3A, 90° section). In this way the 4-fold ncs axes along the unit cell vectors are generated. The combination of the crystallographic symmetry with the icosahedral symmetry of the particle results in four 5-fold axes coinciding roughly with the (110) direction. This leads to dominant rotation function peaks in the κ = 72° section (Fig. 3A). These prominent rotation function peaks hide the icosahedral symmetry of the VLP very efficiently.

The refined FCV VLP structure differed by 4.0 Å rms from the model-built structure and by 8.3 Å rms from the pseudo-atomic model obtained from EM reconstruction which suffered from the absence of an independent refinement of the S and P domains, furthermore the presence of the NTA domain. The insufficient accuracy of the model together with the domination of the rotation function by the crystallographic symmetry and its interplay with the icosahedral symmetry explain certainly the failure of direct molecular replacement.

**Fig 5:**
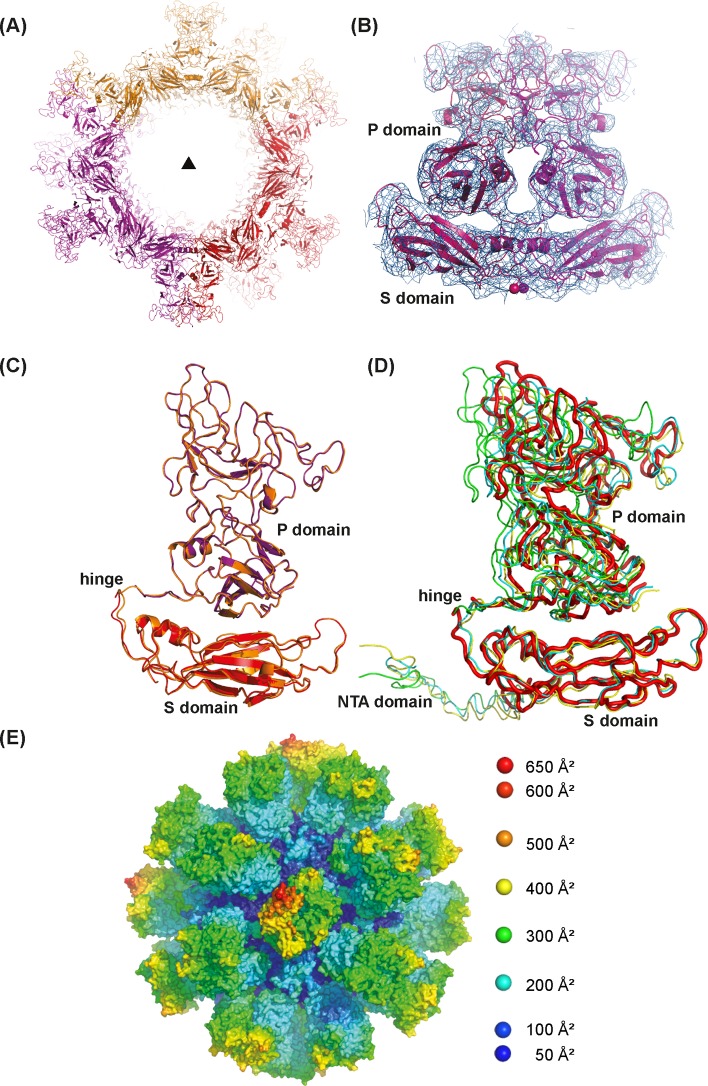
Structure of the FCV VP1 VLP. (A) The 8 Å electron density of a map after phase improvement by 20-fold ncs. Initial phases were based on the rigid-body refined model of the FCV VLP. The electron density is contoured at 0.75 σ level and shown around one of the VP1 dimers. Spheres (arrow) mark the N-terminal end of the visible electron density at res. 161. (B) Monomer of VP1, the building block of the VLP. The S domain forming the protein shell is shown in red, the P domain forming the dimeric spikes is shown in violet, an arrow shows N-terminal end of the visible electron density. The monomer structure obtained after restrained refinement (orange, see S1 Table) is shown superposed (monomer S, 0.45 Å rms at the level of the Cα atoms). (C) Section through the sub-viral particle looking down the 3-fold axis of the (111) direction (triangle). Subunits are colored according to the crystallographic asymmetric unit they belong to. (D) Superposition of the S domains from the 3 independent VP1 monomers from the T = 3 FCV structure (pdb 3m8l, monomer A: yellow; B: cyan; C: green) onto the monomer from the T = 1 VLP (red). The different orientations of the P domain resulting from the hinge movement become evident. As the T = 1 structure is based on monomer C, its S domain matches the T = 1 structure perfectly. (E) Surface of the VLP after restrained refinement (see S1 Table) colored according to the isotropic temperature factor obtained after tlsanl [34] ranging from 45 Å^2^ (blue) to 640 Å^2^ (red).

Despite the low resolution of the data, the high redundancy of the information allowed to calculate an experimental electron density map which agreed with the known structure of FCV VP1 after rigid-body refinement (Fig. 5A). Only a small number of degrees of freedom were introduced in this process (Table 1). R_cryst_ and R_free_ stayed at values which are rather high but not unprecedented [33]. R-factor and model quality could be improved considerably using restrained refinement imposing tight ncs symmetry combined with TLS refinement [34] of the P domains. This decreased the R_cryst_ from 0.427 to 0.300 and R_free_ from 0.448 to 0.371 and led to better geometry and side chain packing of the model (S1 Table). Still, as Cα atom positions are within 0.45 Å rms from the rigid-body refined structure (Fig. 5B) and deviations from icosahedral symmetry remain negligible, the unique chain of the rigid-body refined model has been deposited in the pdb and is used for further analysis.

### VP1 VLP structure

VP1 forms a closely packed shell (Fig. 5C) without any significant openings. The particle has an outer biggest dimension of 295 Å at the position of the spikes, whereas the shell extends only to a diameter of 185 Å. The inner cavity has a diameter of 125 Å. In the crystal structure electron density appears to start at residue 161 close to the limit between the NTA and the S domain (Fig. 5B and S2A Fig.). There is no indication that the structures of the S and the P domains differ from the corresponding ones from the T = 3 crystal structure of the viral particle. But in order to adapt to the T = 1 shell, the relative positions of the S and P domains change from the ones observed in the T = 3 structure (Fig. 5D). When the S domains of the non-equivalent trimer from the facet of the T = 3 structure of the FCV capsid (chains A, B and C in pdb entry 3m8l) are superposed onto the S domains of the strict trimer of the T = 1 VLP, the P domains of chain C, the one used for model building, rotates by roughly 13° around the 2-fold axis of the spike. The Cα-atom of hinge residue 331, actually located close to the 2-fold axis, moves by 4.0 Å. The corresponding rotations and translations for the A and B chains are respectively: 21.4° and 2.0 Å and 17.5° and 3.3 Å.

It is likely that the flexibility of the P domains relative to the S domains induces the disorder of the spikes obvious from the temperature factor distribution in the model (Fig. 5E). This disorder limits probably also the resolution of the EM reconstruction which otherwise agrees well with the crystal structure (S2B Fig.). A cryo-EM reconstruction of RHDV T = 1 subviral particles showed a similar disorder of the spikes [35].

### VLP formation

In the same way as the T = 3 shell, the T = 1 shell is composed of VP1 molecules arranged as dimers, trimers and pentamers [36]. The T = 3 particle contains additional hexamers with pseudo 6-fold axes which are located on the 3-fold axes of the icosahedron, whereas its trimer contacts are located in the center of the facets formed by three non-equivalent VP1 molecules. The VP1 dimers form the spikes. A comparison of these different oligomers in the T = 1 and the T = 3 structures confirm the conservation of the contacts within the oligomers. Equivalent oligomers can be superposed at the level of the S domain with rms deviations between 1.7 and 2.4 Å (S3 Fig.) where the assembly around the 2-fold and 5-fold axes is particularly well conserved. The structural adaptation to the two types of particles involves a hinge movement between the VP1 S and P domains (Fig. 5B and 5D) resulting in a slight rotation of the spike around its 2-fold axis which is combined with a translation of the P domain relative to the S domain. The same flexibility is used in order create the 3 non-equivalent subunits forming the facet of the T = 3 VLP where the relative orientations of the P domains differ actually by up to 12.3° and involve translations up to 3.1 Å at the level of hinge residue 331 (Fig. 5D). It is likely that the T = 3 particle is stabilized further by the N-terminal interlocking arms [1] which are absent from the T = 1 VLP structure.

The question whether the proteolysis of the NTA domain is the reason for the preferred assembly into T = 1 particles, or whether it is rather a consequence cannot be answered. Bárcena and co-workers observed that N-terminal truncation mutants of RHDV expressed in insect cells assembled into T = 1 particles [35], but for Norwalk virus N-terminal truncations did not lead to the formation of small capsids [36]. T = 1 and T = 3 VLPs were observed simultaneously when NV VP1 was expressed [37] and it could be shown that one form of the VLP could be transformed into the other one after dissociation suggesting that the same peptide chains are present in both types of particles. An increased susceptibility to proteolysis of small norovirus VLPs was observed which could be a consequence of the absence of an ordered conformation of the NTA domain in the small particles [37]. As there are no significant openings of the shell and the N-termini are located *a priori* inside calicivirus capsid, it is likely that proteolysis of FCV VP1 takes place either before assembly or during transient disassembly of the VLPs. Indeed, they had a tendency to disaggregate when observed in electron microscopy where a slightly acidic pH was favorable for their stability.

## Conclusions

The conservation of the arrangement of the VP1 capsomers around the 2-fold, the 5-fold and the 3-fold icosahedral axes between the native T = 3 virus particles and the T = 1 VLPs shows that the antigenicity of the protein is conserved and that the use of the T = 1 particles for vaccination is a pertinent approach. Still the stability of the T = 1 VLPs may be reduced due to the absence of the intermolecular interaction of the NTA arms. The T = 1 VLP structure could be used to design strategies for a stabilization of the particle for vaccine development.

## Supporting Information

S1 Table Statistics of a restrained refinement with refmac5. (PDF)

S1 Fig Fourier shell correlation plot of the cryo-EM reconstruction of the T = 1 FCV VLP. (PDF)

S2 Fig Stereoviews of electron density of FCV VLPs. (A) Electron density of one spike dimer from Fig. 5A. The electron density of a map calculated after phase improvement by 20-fold ncs is contoured at 0.75 σ. The spheres mark the N-terminal end of the visible electron density. (B) Low-resolution envelope of a reconstruction from cryo-EM images and a ribbon representation of the final pseudo-atomic model of FCV VLP in icosahedral standard orientation.
(PDF)

S3 Fig Comparison of the subunit contacts in the T = 1 VLP (red and violet tints) and the T = 3 viral capsid (yellow and green colors). The alignment used Cα atoms from the S domains of all subunits forming the corresponding oligomer contact. The rms deviation of the equivalent Cα atom positions in the corresponding oligomers is indicated. (A) Pentamer contact seen from the inside of the shell, (B) side view of the dimer contact, (C) trimer contact seen from the inside of the shell. In the T = 3 shell, the 3 different monomers forming one facet are related by a quasi 3-fold axis and differ in their conformation (see Fig. 5D).
(PDF)
