# Supplementary material for: Structure Determination of Feline Calicivirus Virus-Like Particles in the Context of a Pseudo-Octahedral Arrangement
Source: PLoS One. 2015 Mar 20;10(3):e0119289. doi: 10.1371/journal.pone.0119289 (PMC4368116; doi:10.1371/journal.pone.0119289)
Supplement: S1 Table — (PDF) [file pone.0119289.s001.pdf]

TABLE S1: Statistics of a restrained refinement with refmac5.

**Resolution range (Å)**	67 - 8.0 (8.2 - 8.0)
**R_{cryst} from refmac5**	0.300 (0.380)
**R_{free} from refmac5**	0.371 (0.437)
**Rms bond lengths**	0.009 Å^2
**Rms bond angles**	1.3°
**Rms deviation of Cα atoms relative to average position**	0.11 Å
**Rms deviation of Cα atoms from rigid body refined structure**	0.45 Å
**Ramachandran plot (Procheck [1]):**	
**Residues in generously allowed regions**	4.6 %
**Residues in disallowed regions**	2.3 %

1. Laskowski RA, Macarthur MW, Moss DS, Thornton JM (1993) Procheck - a Program to Check the Stereochemical Quality of Protein Structures. J Appl Crystallogr 26: 283–291.
