# Supplementary figures and images for: Structure Determination of Feline Calicivirus Virus-Like Particles in the Context of a Pseudo-Octahedral Arrangement
Source: PLoS One. 2015 Mar 20;10(3):e0119289. doi: 10.1371/journal.pone.0119289 (PMC4368116; doi:10.1371/journal.pone.0119289)

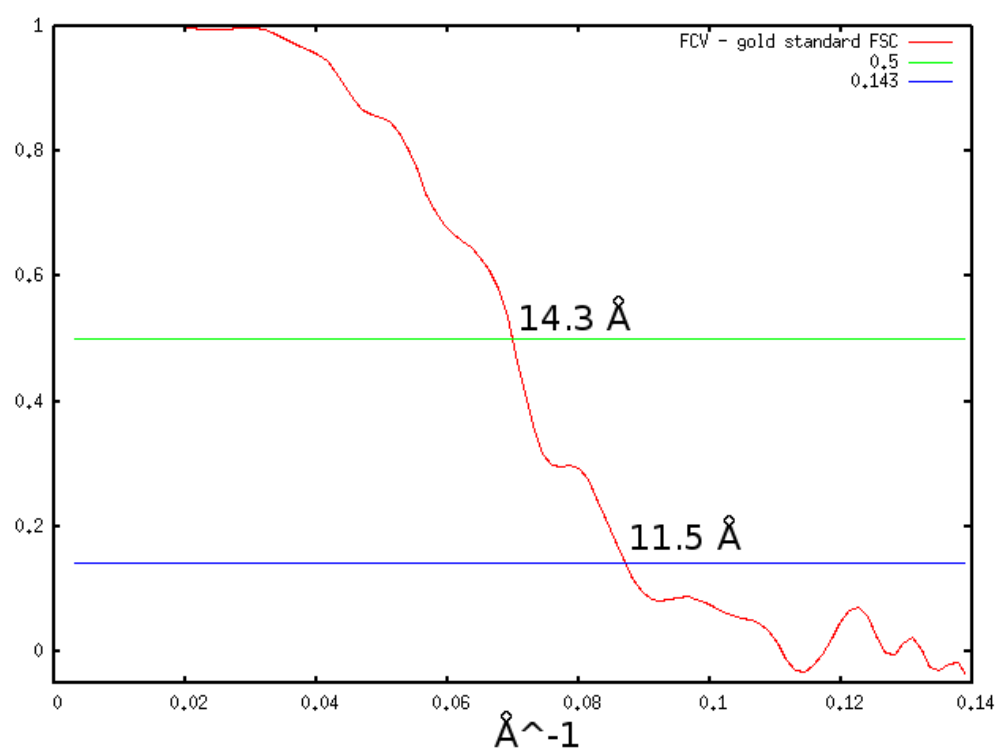

**Figure S1. Fourier shell correlation plot of the cryo-EM reconstruction of the T=1 FCV VLP.**

Supplement: S1 Fig — (PDF) [file pone.0119289.s002.pdf]
