# Supplementary material for: Structure Determination of Feline Calicivirus Virus-Like Particles in the Context of a Pseudo-Octahedral Arrangement
Source: PLoS One. 2015 Mar 20;10(3):e0119289. doi: 10.1371/journal.pone.0119289 (PMC4368116; doi:10.1371/journal.pone.0119289)
Supplement: S2 Fig — (A) Electron density of one spike dimer from Fig. 5A. The electron density of a map calculated after phase improvement by 20-fold ncs is contoured at 0.75 σ. The spheres mark the N-terminal end of the visible electron density. (B) Low-resolution envelope of a reconstruction from cryo-EM images and a ribbon representation of the final pseudo-atomic model of FCV VLP in icosahedral standard orientation. (PDF) [file pone.0119289.s003.pdf]

(A)

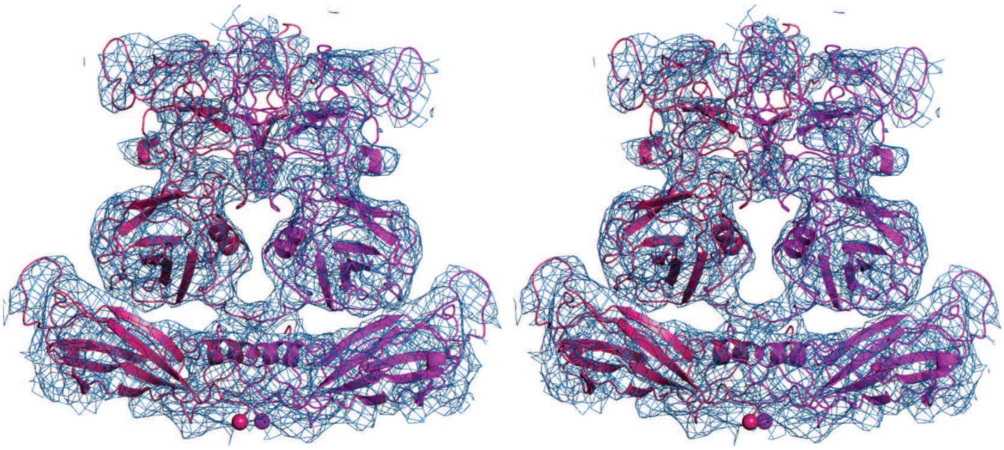

(B)

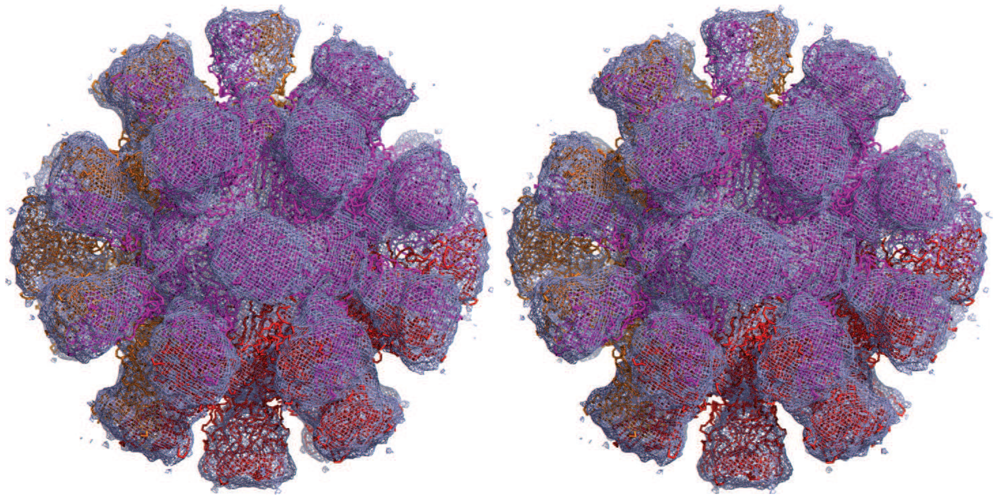

Figure S2. Stereoviews of electron density of FCV VLPs. (A) Electron density of one spike dimer from Figure 5A. The electron density of a map calculated after phase improvement by 20-fold ncs is contoured at 0.75 σ. The spheres mark the N-terminal end of the visible electron density. (B) Low-resolution envelope of a reconstruction from cryo-EM images and a ribbon representation of the final pseudo-atomic model of FCV VLP in icosahedral standard orientation.
