# Supplementary material for: Structure Determination of Feline Calicivirus Virus-Like Particles in the Context of a Pseudo-Octahedral Arrangement
Source: PLoS One. 2015 Mar 20;10(3):e0119289. doi: 10.1371/journal.pone.0119289 (PMC4368116; doi:10.1371/journal.pone.0119289)
Supplement: S3 Fig — The alignment used Cα atoms from the S domains of all subunits forming the corresponding oligomer contact. The rms deviation of the equivalent Cα atom positions in the corresponding oligomers is indicated. (A) Pentamer contact seen from the inside of the shell, (B) side view of the dimer contact, (C) trimer contact seen from the inside of the shell. In the T = 3 shell, the 3 different monomers forming one facet are related by a quasi 3-fold axis and differ in their conformation (see Fig. 5D). (PDF) [file pone.0119289.s004.pdf]

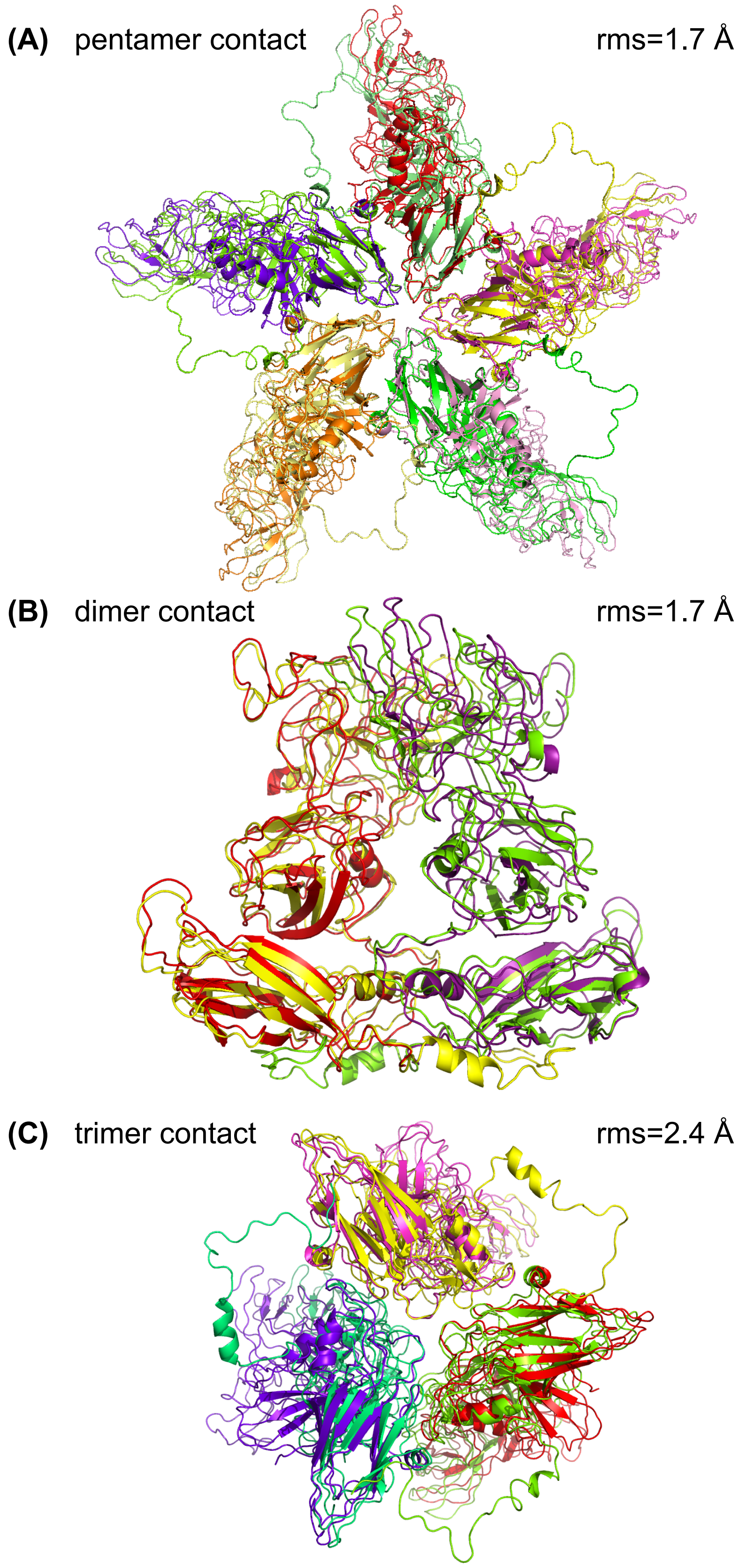

Figure S3. Comparison of the subunit contacts in the T=1 VLP (red and violet tints) and the T=3 viral capsid (yellow and green colors). The alignment used Cα atoms from the S domains of all subunits forming the corresponding oligomer contact. The rms deviation of the equivalent Cα atom positions in the corresponding oligomers is indicated. (A) Pentamer contact seen from the inside of the shell, (B) side view of the dimer contact, (C) trimer contact seen from the inside of the shell. In the T=3 shell, the 3 different monomers forming one facet are related by a quasi 3-fold axis and differ in their conformation (see Figure 5D).
